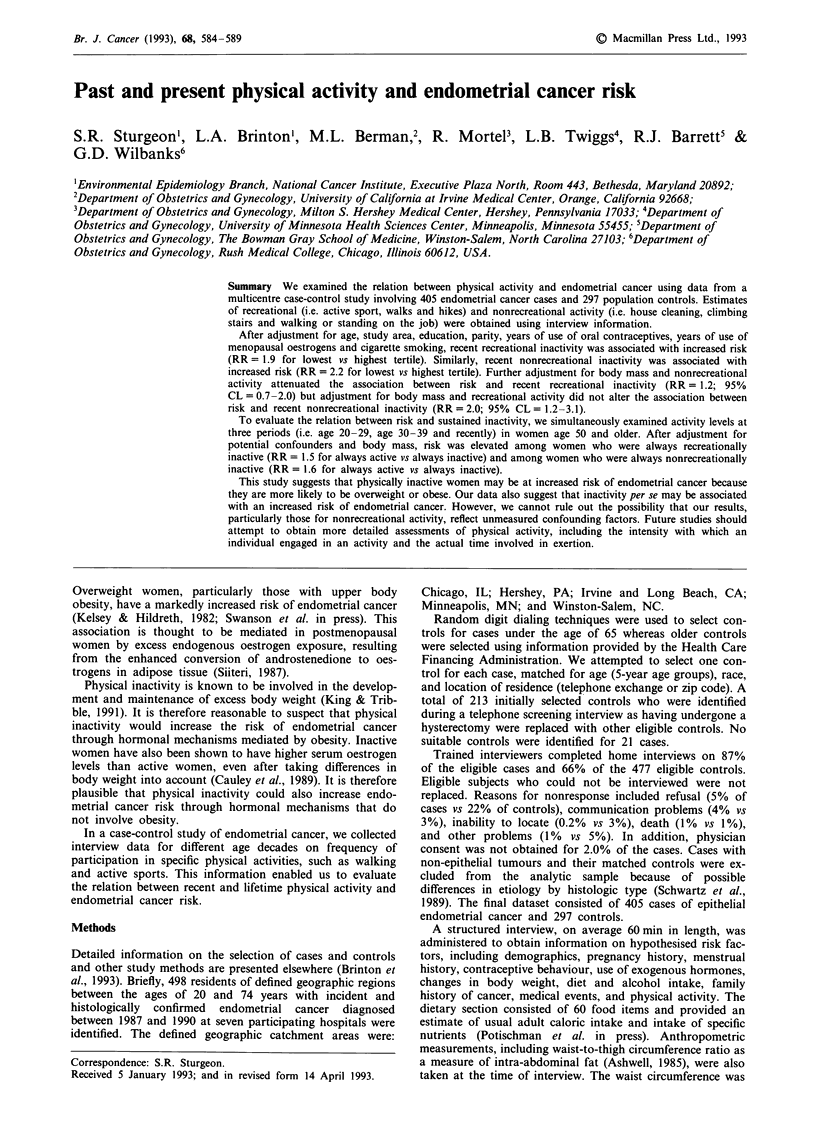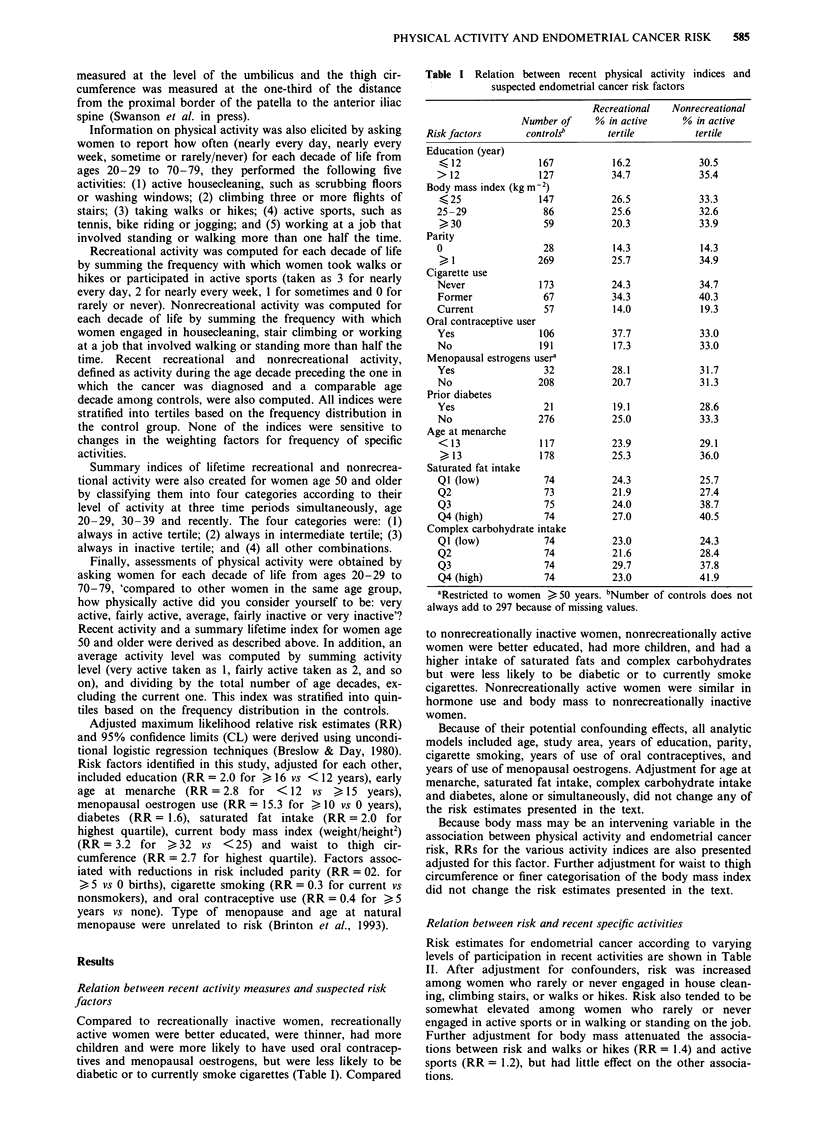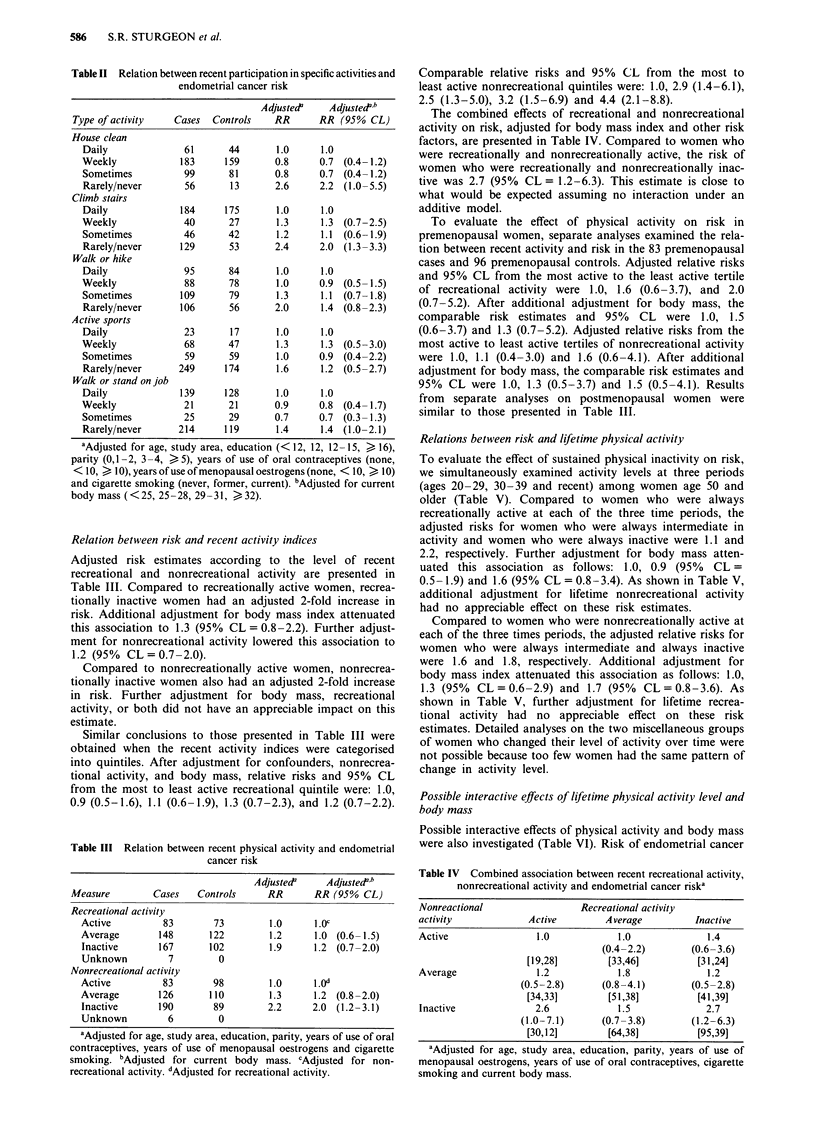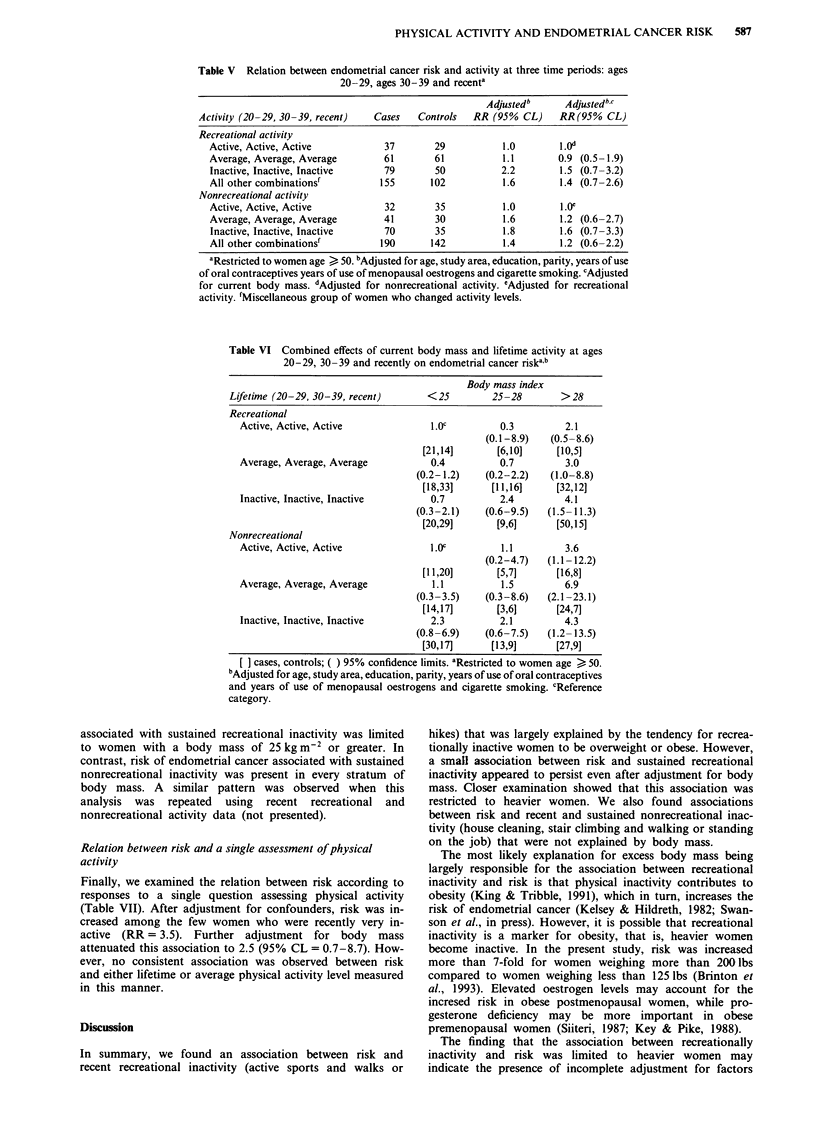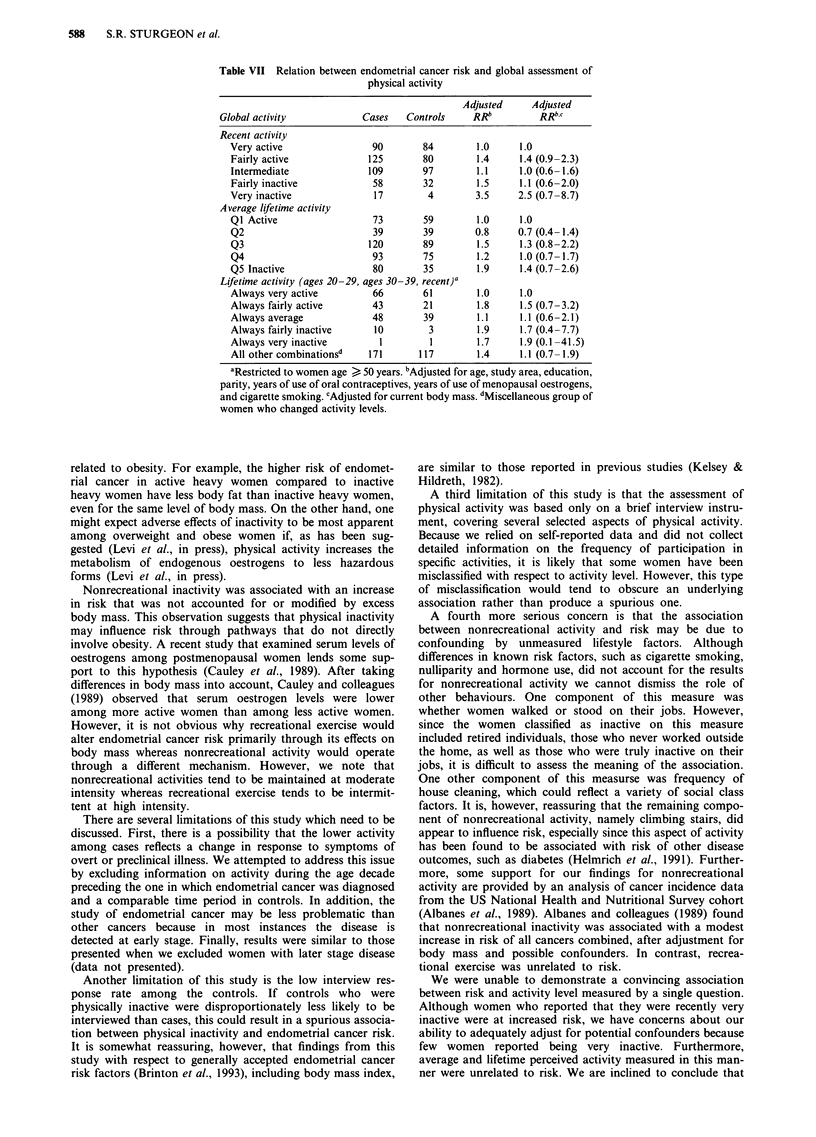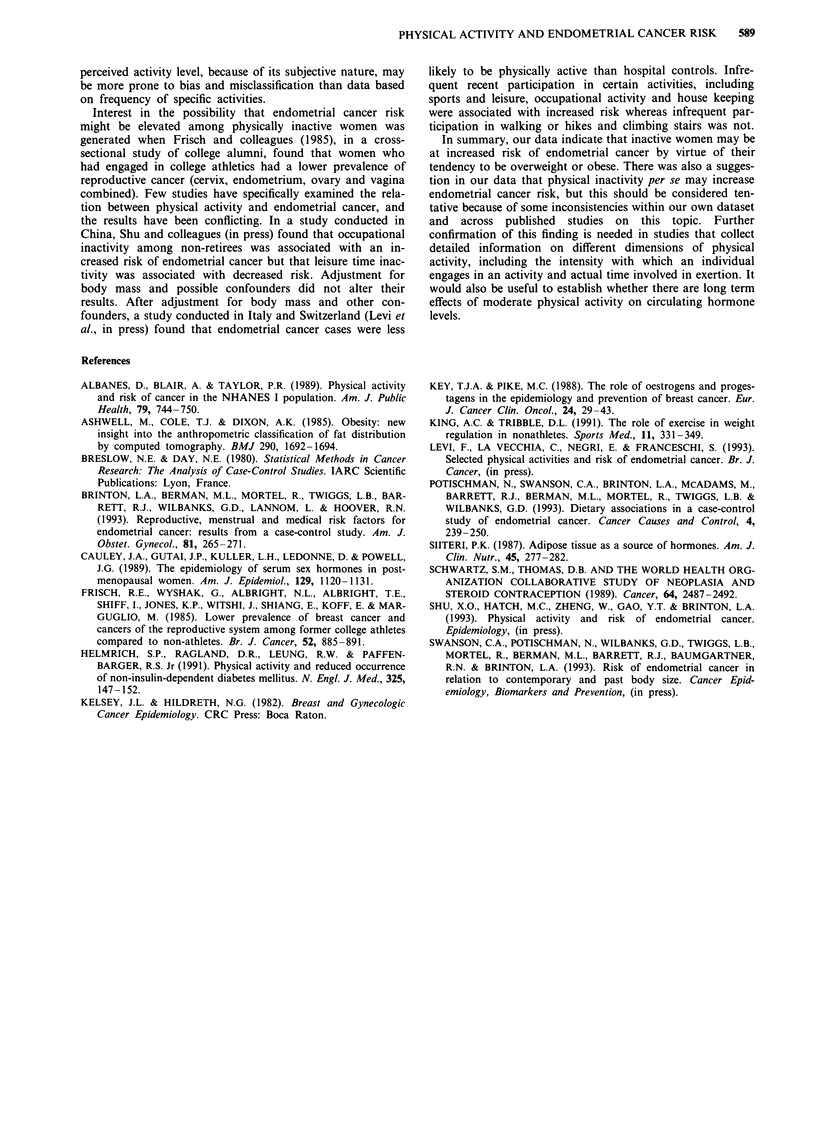# Past and present physical activity and endometrial cancer risk.

**DOI:** 10.1038/bjc.1993.390

**Published:** 1993-09

**Authors:** S. R. Sturgeon, L. A. Brinton, M. L. Berman, R. Mortel, L. B. Twiggs, R. J. Barrett, G. D. Wilbanks

**Affiliations:** Environmental Epidemiology Branch, National Cancer Institute, Bethesda, Maryland 20892.

## Abstract

We examined the relation between physical activity and endometrial cancer using data from a multicentre case-control study involving 405 endometrial cancer cases and 297 population controls. Estimates of recreational (i.e. active sport, walks and hikes) and nonrecreational activity (i.e. house cleaning, climbing stairs and walking or standing on the job) were obtained using interview information. After adjustment for age, study area, education, parity, years of use of oral contraceptives, years of use of menopausal oestrogens and cigarette smoking, recent recreational inactivity was associated with increased risk (RR = 1.9 for lowest vs highest tertile). Similarly, recent nonrecreational inactivity was associated with increased risk (RR = 2.2 for lowest vs highest tertile). Further adjustment for body mass and nonrecreational activity attenuated the association between risk and recent recreational inactivity (RR = 1.2; 95% CL = 0.7-2.0) but adjustment for body mass and recreational activity did not alter the association between risk and recent nonrecreational inactivity (RR = 2.0; 95% CL = 1.2-3.1). To evaluate the relation between risk and sustained inactivity, we simultaneously examined activity levels at three periods (RR i.e. age 20-29, age 30-39 and recently) in women age 50 and older. After adjustment for potential confounders and body mass, risk was elevated among women who were always recreationally inactive (RR = 1.5 for always active vs always inactive) and among women who were always nonrecreationally inactive (RR = 1.6 for always active vs always inactive). This study suggests that physically inactive women may be at increased risk of endometrial cancer because they are more likely to be overweight or obese. Our data also suggest that inactivity per se may be associated with an increased risk of endometrial cancer. However, we cannot rule out the possibility that our results, particularly those for nonrecreational activity, reflect unmeasured confounding factors. Future studies should attempt to obtain more detailed assessments of physical activity, including the intensity with which an individual engaged in an activity and the actual time involved in exertion.


Br. J. Cancer (1993), 68, 584–589 © Macmillan Press Ltd., 1993

### Past and present physical activity and endometrial cancer risk

S.R. Sturgeon[1], L.A. Brinton[1], M.L. Berman,[2], R. Mortel[3], L.B. Twiggs[4], R.J. Barrett[5] & G.D. Wilbanks[6]

[1]*Environmental Epidemiology Branch, National Cancer Institute, Executive Plaza North, Room 443, Bethesda, Maryland 20892;* [2]*Department of Obstetrics and Gynecology, University of California at Irvine Medical Center, Orange, California 92668;* [3]*Department of Obstetrics and Gynecology, Milton S. Hershey Medical Center, Hershey, Pennsylvania 17033;* [4]*Department of Obstetrics and Gynecology, University of Minnesota Health Sciences Center, Minneapolis, Minnesota 55455;* [5]*Department of Obstetrics and Gynecology, The Bowman Gray School of Medicine, Winston-Salem, North Carolina 27103;* [6]*Department of Obstetrics and Gynecology, Rush Medical College, Chicago, Illinois 60612, USA.*

**Summary** We examined the relation between physical activity and endometrial cancer using data from a multicentre case-control study involving 405 endometrial cancer cases and 297 population controls. Estimates of recreational (i.e. active sport, walks and hikes) and nonrecreational activity (i.e. house cleaning, climbing stairs and walking or standing on the job) were obtained using interview information.

After adjustment for age, study area, education, parity, years of use of oral contraceptives, years of use of menopausal oestrogens and cigarette smoking, recent recreational inactivity was associated with increased risk (RR = 1.9 for lowest *vs* highest tertile). Similarly, recent nonrecreational inactivity was associated with increased risk (RR = 2.2 for lowest *vs* highest tertile). Further adjustment for body mass and nonrecreational activity attenuated the association between risk and recent recreational inactivity (RR = 1.2; 95% CL = 0.7–2.0) but adjustment for body mass and recreational activity did not alter the association between risk and recent nonrecreational inactivity (RR = 2.0; 95% CL = 1.2–3.1).

To evaluate the relation between risk and sustained inactivity, we simultaneously examined activity levels at three periods (i.e. age 20–29, age 30–39 and recently) in women age 50 and older. After adjustment for potential confounders and body mass, risk was elevated among women who were always recreationally inactive (RR = 1.5 for always active *vs* always inactive) and among women who were always nonrecreationally inactive (RR = 1.6 for always active *vs* always inactive).

This study suggests that physically inactive women may be at increased risk of endometrial cancer because they are more likely to be overweight or obese. Our data also suggest that inactivity *per se* may be associated with an increased risk of endometrial cancer. However, we cannot rule out the possibility that our results, particularly those for nonrecreational activity, reflect unmeasured confounding factors. Future studies should attempt to obtain more detailed assessments of physical activity, including the intensity with which an individual engaged in an activity and the actual time involved in exertion.

Overweight women, particularly those with upper body obesity, have a markedly increased risk of endometrial cancer (Kelsey & Hildreth, 1982; Swanson *et al.* in press). This association is thought to be mediated in postmenopausal women by excess endogenous oestrogen exposure, resulting from the enhanced conversion of androstenedione to oestrogens in adipose tissue (Siiteri, 1987).

Physical inactivity is known to be involved in the development and maintenance of excess body weight (King & Tribble, 1991). It is therefore reasonable to suspect that physical inactivity would increase the risk of endometrial cancer through hormonal mechanisms mediated by obesity. Inactive women have also been shown to have higher serum oestrogen levels than active women, even after taking differences in body weight into account (Cauley *et al.*, 1989). It is therefore plausible that physical inactivity could also increase endometrial cancer risk through hormonal mechanisms that do not involve obesity.

In a case-control study of endometrial cancer, we collected interview data for different age decades on frequency of participation in specific physical activities, such as walking and active sports. This information enabled us to evaluate the relation between recent and lifetime physical activity and endometrial cancer risk.

### Methods

Detailed information on the selection of cases and controls and other study methods are presented elsewhere (Brinton *et al.*, 1993). Briefly, 498 residents of defined geographic regions between the ages of 20 and 74 years with incident and histologically confirmed endometrial cancer diagnosed between 1987 and 1990 at seven participating hospitals were identified. The defined geographic catchment areas were: Chicago, IL; Hershey, PA; Irvine and Long Beach, CA; Minneapolis, MN; and Winston-Salem, NC.

Random digit dialing techniques were used to select controls for cases under the age of 65 whereas older controls were selected using information provided by the Health Care Financing Administration. We attempted to select one control for each case, matched for age (5-year age groups), race, and location of residence (telephone exchange or zip code). A total of 213 initially selected controls who were identified during a telephone screening interview as having undergone a hysterectomy were replaced with other eligible controls. No suitable controls were identified for 21 cases.

Trained interviewers completed home interviews on 87% of the eligible cases and 66% of the 477 eligible controls. Eligible subjects who could not be interviewed were not replaced. Reasons for nonresponse included refusal (5% of cases *vs* 22% of controls), communication problems (4% *vs* 3%), inability to locate (0.2% *vs* 3%), death (1% *vs* 1%), and other problems (1% *vs* 5%). In addition, physician consent was not obtained for 2.0% of the cases. Cases with non-epithelial tumours and their matched controls were excluded from the analytic sample because of possible differences in etiology by histologic type (Schwartz *et al.*, 1989). The final dataset consisted of 405 cases of epithelial endometrial cancer and 297 controls.

A structured interview, on average 60 min in length, was administered to obtain information on hypothesised risk factors, including demographics, pregnancy history, menstrual history, contraceptive behaviour, use of exogenous hormones, changes in body weight, diet and alcohol intake, family history of cancer, medical events, and physical activity. The dietary section consisted of 60 food items and provided an estimate of usual adult caloric intake and intake of specific nutrients (Potischman *et al.* in press). Anthropometric measurements, including waist-to-thigh circumference ratio as a measure of intra-abdominal fat (Ashwell, 1985), were also taken at the time of interview. The waist circumference was

Correspondence: S.R. Sturgeon.
Received 5 January 1993; and in revised form 14 April 1993.

measured at the level of the umbilicus and the thigh circumference was measured at the one-third of the distance from the proximal border of the patella to the anterior iliac spine (Swanson *et al.* in press).

Information on physical activity was also elicited by asking women to report how often (nearly every day, nearly every week, sometime or rarely/never) for each decade of life from ages 20–29 to 70–79, they performed the following five activities: (1) active housecleaning, such as scrubbing floors or washing windows; (2) climbing three or more flights of stairs; (3) taking walks or hikes; (4) active sports, such as tennis, bike riding or jogging; and (5) working at a job that involved standing or walking more than one half the time.

Recreational activity was computed for each decade of life by summing the frequency with which women took walks or hikes or participated in active sports (taken as 3 for nearly every day, 2 for nearly every week, 1 for sometimes and 0 for rarely or never). Nonrecreational activity was computed for each decade of life by summing the frequency with which women engaged in housecleaning, stair climbing or working at a job that involved walking or standing more than half the time. Recent recreational and nonrecreational activity, defined as activity during the age decade preceding the one in which the cancer was diagnosed and a comparable age decade among controls, were also computed. All indices were stratified into tertiles based on the frequency distribution in the control group. None of the indices were sensitive to changes in the weighting factors for frequency of specific activities.

Summary indices of lifetime recreational and nonrecreational activity were also created for women age 50 and older by classifying them into four categories according to their level of activity at three time periods simultaneously, age 20–29, 30–39 and recently. The four categories were: (1) always in active tertile; (2) always in intermediate tertile; (3) always in inactive tertile; and (4) all other combinations.

Finally, assessments of physical activity were obtained by asking women for each decade of life from ages 20–29 to 70–79, 'compared to other women in the same age group, how physically active did you consider yourself to be: very active, fairly active, average, fairly inactive or very inactive'? Recent activity and a summary lifetime index for women age 50 and older were derived as described above. In addition, an average activity level was computed by summing activity level (very active taken as 1, fairly active taken as 2, and so on), and dividing by the total number of age decades, excluding the current one. This index was stratified into quintiles based on the frequency distribution in the controls.

Adjusted maximum likelihood relative risk estimates (RR) and 95% confidence limits (CL) were derived using unconditional logistic regression techniques (Breslow & Day, 1980). Risk factors identified in this study, adjusted for each other, included education (RR = 2.0 for ⩾16 *vs* <12 years), early age at menarche (RR = 2.8 for <12 *vs* ⩾15 years), menopausal oestrogen use (RR = 15.3 for ⩾10 *vs* 0 years), diabetes (RR = 1.6), saturated fat intake (RR = 2.0 for highest quartile), current body mass index (weight/height$^2$) (RR = 3.2 for ⩾32 *vs* <25) and waist to thigh circumference (RR = 2.7 for highest quartile). Factors associated with reductions in risk included parity (RR = 02. for ⩾5 *vs* 0 births), cigarette smoking (RR = 0.3 for current *vs* nonsmokers), and oral contraceptive use (RR = 0.4 for ⩾5 years *vs* none). Type of menopause and age at natural menopause were unrelated to risk (Brinton *et al.*, 1993).

### Results

### *Relation between recent activity measures and suspected risk factors*

Compared to recreationally inactive women, recreationally active women were better educated, were thinner, had more children and were more likely to have used oral contraceptives and menopausal oestrogens, but were less likely to be diabetic or to currently smoke cigarettes (Table I). Compared to nonrecreationally inactive women, nonrecreationally active women were better educated, had more children, and had a higher intake of saturated fats and complex carbohydrates but were less likely to be diabetic or to currently smoke cigarettes. Nonrecreationally active women were similar in hormone use and body mass to nonrecreationally inactive women.

**Table I** Relation between recent physical activity indices and suspected endometrial cancer risk factors

| *Risk factors* | *Number of controls*[b] | *Recreational % in active tertile* | *Nonrecreational % in active tertile* |
|---|---|---|---|
| Education (year) | | | |
| ⩽12 | 167 | 16.2 | 30.5 |
| >12 | 127 | 34.7 | 35.4 |
| Body mass index (kg m$^{-2}$) | | | |
| ⩽25 | 147 | 26.5 | 33.3 |
| 25–29 | 86 | 25.6 | 32.6 |
| ⩾30 | 59 | 20.3 | 33.9 |
| Parity | | | |
| 0 | 28 | 14.3 | 14.3 |
| ⩾1 | 269 | 25.7 | 34.9 |
| Cigarette use | | | |
| Never | 173 | 24.3 | 34.7 |
| Former | 67 | 34.3 | 40.3 |
| Current | 57 | 14.0 | 19.3 |
| Oral contraceptive user | | | |
| Yes | 106 | 37.7 | 33.0 |
| No | 191 | 17.3 | 33.0 |
| Menopausal estrogens user[a] | | | |
| Yes | 32 | 28.1 | 31.7 |
| No | 208 | 20.7 | 31.3 |
| Prior diabetes | | | |
| Yes | 21 | 19.1 | 28.6 |
| No | 276 | 25.0 | 33.3 |
| Age at menarche | | | |
| <13 | 117 | 23.9 | 29.1 |
| ⩾13 | 178 | 25.3 | 36.0 |
| Saturated fat intake | | | |
| Q1 (low) | 74 | 24.3 | 25.7 |
| Q2 | 73 | 21.9 | 27.4 |
| Q3 | 75 | 24.0 | 38.7 |
| Q4 (high) | 74 | 27.0 | 40.5 |
| Complex carbohydrate intake | | | |
| Q1 (low) | 74 | 23.0 | 24.3 |
| Q2 | 74 | 21.6 | 28.4 |
| Q3 | 74 | 29.7 | 37.8 |
| Q4 (high) | 74 | 23.0 | 41.9 |

[a]Restricted to women ⩾50 years. [b]Number of controls does not always add to 297 because of missing values.

Because of their potential confounding effects, all analytic models included age, study area, years of education, parity, cigarette smoking, years of use of oral contraceptives, and years of use of menopausal oestrogens. Adjustment for age at menarche, saturated fat intake, complex carbohydrate intake and diabetes, alone or simultaneously, did not change any of the risk estimates presented in the text.

Because body mass may be an intervening variable in the association between physical activity and endometrial cancer risk, RRs for the various activity indices are also presented adjusted for this factor. Further adjustment for waist to thigh circumference or finer categorisation of the body mass index did not change the risk estimates presented in the text.

### *Relation between risk and recent specific activities*

Risk estimates for endometrial cancer according to varying levels of participation in recent activities are shown in Table II. After adjustment for confounders, risk was increased among women who rarely or never engaged in house cleaning, climbing stairs, or walks or hikes. Risk also tended to be somewhat elevated among women who rarely or never engaged in active sports or in walking or standing on the job. Further adjustment for body mass attenuated the associations between risk and walks or hikes (RR = 1.4) and active sports (RR = 1.2), but had little effect on the other associations.

**Table II** Relation between recent participation in specific activities and endometrial cancer risk

| Type of activity | Cases | Controls | Adjusted[a] RR | Adjusted[a,b] RR (95% CL) |
|---|---|---|---|---|
| *House clean* | | | | |
| Daily | 61 | 44 | 1.0 | 1.0 |
| Weekly | 183 | 159 | 0.8 | 0.7 (0.4–1.2) |
| Sometimes | 99 | 81 | 0.8 | 0.7 (0.4–1.2) |
| Rarely/never | 56 | 13 | 2.6 | 2.2 (1.0–5.5) |
| *Climb stairs* | | | | |
| Daily | 184 | 175 | 1.0 | 1.0 |
| Weekly | 40 | 27 | 1.3 | 1.3 (0.7–2.5) |
| Sometimes | 46 | 42 | 1.2 | 1.1 (0.6–1.9) |
| Rarely/never | 129 | 53 | 2.4 | 2.0 (1.3–3.3) |
| *Walk or hike* | | | | |
| Daily | 95 | 84 | 1.0 | 1.0 |
| Weekly | 88 | 78 | 1.0 | 0.9 (0.5–1.5) |
| Sometimes | 109 | 79 | 1.3 | 1.1 (0.7–1.8) |
| Rarely/never | 106 | 56 | 2.0 | 1.4 (0.8–2.3) |
| *Active sports* | | | | |
| Daily | 23 | 17 | 1.0 | 1.0 |
| Weekly | 68 | 47 | 1.3 | 1.3 (0.5–3.0) |
| Sometimes | 59 | 59 | 1.0 | 0.9 (0.4–2.2) |
| Rarely/never | 249 | 174 | 1.6 | 1.2 (0.5–2.7) |
| *Walk or stand on job* | | | | |
| Daily | 139 | 128 | 1.0 | 1.0 |
| Weekly | 21 | 21 | 0.9 | 0.8 (0.4–1.7) |
| Sometimes | 25 | 29 | 0.7 | 0.7 (0.3–1.3) |
| Rarely/never | 214 | 119 | 1.4 | 1.4 (1.0–2.1) |

[a]Adjusted for age, study area, education (<12, 12, 12–15, ⩾16), parity (0,1–2, 3–4, ⩾5), years of use of oral contraceptives (none, <10, ⩾10), years of use of menopausal oestrogens (none, <10, ⩾10) and cigarette smoking (never, former, current). [b]Adjusted for current body mass (<25, 25–28, 29–31, ⩾32).

### *Relation between risk and recent activity indices*

Adjusted risk estimates according to the level of recent recreational and nonrecreational activity are presented in Table III. Compared to recreationally active women, recreationally inactive women had an adjusted 2-fold increase in risk. Additional adjustment for body mass index attenuated this association to 1.3 (95% CL = 0.8–2.2). Further adjustment for nonrecreational activity lowered this association to 1.2 (95% CL = 0.7–2.0).

Compared to nonrecreationally active women, nonrecreationally inactive women also had an adjusted 2-fold increase in risk. Further adjustment for body mass, recreational activity, or both did not have an appreciable impact on this estimate.

Similar conclusions to those presented in Table III were obtained when the recent activity indices were categorised into quintiles. After adjustment for confounders, nonrecreational activity, and body mass, relative risks and 95% CL from the most to least active recreational quintile were: 1.0, 0.9 (0.5–1.6), 1.1 (0.6–1.9), 1.3 (0.7–2.3), and 1.2 (0.7–2.2).

**Table III** Relation between recent physical activity and endometrial cancer risk

| Measure | Cases | Controls | Adjusted[a] RR | Adjusted[a,b] RR (95% CL) |
|---|---|---|---|---|
| *Recreational activity* | | | | |
| Active | 83 | 73 | 1.0 | 1.0[c] |
| Average | 148 | 122 | 1.2 | 1.0 (0.6–1.5) |
| Inactive | 167 | 102 | 1.9 | 1.2 (0.7–2.0) |
| Unknown | 7 | 0 | | |
| *Nonrecreational activity* | | | | |
| Active | 83 | 98 | 1.0 | 1.0[d] |
| Average | 126 | 110 | 1.3 | 1.2 (0.8–2.0) |
| Inactive | 190 | 89 | 2.2 | 2.0 (1.2–3.1) |
| Unknown | 6 | 0 | | |

[a]Adjusted for age, study area, education, parity, years of use of oral contraceptives, years of use of menopausal oestrogens and cigarette smoking. [b]Adjusted for current body mass. [c]Adjusted for nonrecreational activity. [d]Adjusted for recreational activity.

Comparable relative risks and 95% CL from the most to least active nonrecreational quintiles were: 1.0, 2.9 (1.4–6.1), 2.5 (1.3–5.0), 3.2 (1.5–6.9) and 4.4 (2.1–8.8).

The combined effects of recreational and nonrecreational activity on risk, adjusted for body mass index and other risk factors, are presented in Table IV. Compared to women who were recreationally and nonrecreationally active, the risk of women who were recreationally and nonrecreationally inactive was 2.7 (95% CL = 1.2–6.3). This estimate is close to what would be expected assuming no interaction under an additive model.

To evaluate the effect of physical activity on risk in premenopausal women, separate analyses examined the relation between recent activity and risk in the 83 premenopausal cases and 96 premenopausal controls. Adjusted relative risks and 95% CL from the most active to the least active tertile of recreational activity were 1.0, 1.6 (0.6–3.7), and 2.0 (0.7–5.2). After additional adjustment for body mass, the comparable risk estimates and 95% CL were 1.0, 1.5 (0.6–3.7) and 1.3 (0.7–5.2). Adjusted relative risks from the most active to least active tertiles of nonrecreational activity were 1.0, 1.1 (0.4–3.0) and 1.6 (0.6–4.1). After additional adjustment for body mass, the comparable risk estimates and 95% CL were 1.0, 1.3 (0.5–3.7) and 1.5 (0.5–4.1). Results from separate analyses on postmenopausal women were similar to those presented in Table III.

### *Relations between risk and lifetime physical activity*

To evaluate the effect of sustained physical inactivity on risk, we simultaneously examined activity levels at three periods (ages 20–29, 30–39 and recent) among women age 50 and older (Table V). Compared to women who were always recreationally active at each of the three time periods, the adjusted risks for women who were always intermediate in activity and women who were always inactive were 1.1 and 2.2, respectively. Further adjustment for body mass attenuated this association as follows: 1.0, 0.9 (95% CL = 0.5–1.9) and 1.6 (95% CL = 0.8–3.4). As shown in Table V, additional adjustment for lifetime nonrecreational activity had no appreciable effect on these risk estimates.

Compared to women who were nonrecreationally active at each of the three times periods, the adjusted relative risks for women who were always intermediate and always inactive were 1.6 and 1.8, respectively. Additional adjustment for body mass index attenuated this association as follows: 1.0, 1.3 (95% CL = 0.6–2.9) and 1.7 (95% CL = 0.8–3.6). As shown in Table V, further adjustment for lifetime recreational activity had no appreciable effect on these risk estimates. Detailed analyses on the two miscellaneous groups of women who changed their level of activity over time were not possible because too few women had the same pattern of change in activity level.

### *Possible interactive effects of lifetime physical activity level and body mass*

Possible interactive effects of physical activity and body mass were also investigated (Table VI). Risk of endometrial cancer

**Table IV** Combined association between recent recreational activity, nonrecreational activity and endometrial cancer risk[a]

| Nonreactional activity | Recreational activity: Active | Average | Inactive |
|---|---|---|---|
| Active | 1.0 | 1.0 | 1.4 |
| | | (0.4–2.2) | (0.6–3.6) |
| | [19,28] | [33,46] | [31,24] |
| Average | 1.2 | 1.8 | 1.2 |
| | (0.5–2.8) | (0.8–4.1) | (0.5–2.8) |
| | [34,33] | [51,38] | [41,39] |
| Inactive | 2.6 | 1.5 | 2.7 |
| | (1.0–7.1) | (0.7–3.8) | (1.2–6.3) |
| | [30,12] | [64,38] | [95,39] |

[a]Adjusted for age, study area, education, parity, years of use of menopausal oestrogens, years of use of oral contraceptives, cigarette smoking and current body mass.

**Table V** Relation between endometrial cancer risk and activity at three time periods: ages 20–29, ages 30–39 and recent[a]

| *Activity (20–29, 30–39, recent)* | *Cases* | *Controls* | *Adjusted[b] RR (95% CL)* | *Adjusted[b,c] RR(95% CL)* |
|---|---|---|---|---|
| *Recreational activity* | | | | |
| Active, Active, Active | 37 | 29 | 1.0 | 1.0[d] |
| Average, Average, Average | 61 | 61 | 1.1 | 0.9 (0.5–1.9) |
| Inactive, Inactive, Inactive | 79 | 50 | 2.2 | 1.5 (0.7–3.2) |
| All other combinations[f] | 155 | 102 | 1.6 | 1.4 (0.7–2.6) |
| *Nonrecreational activity* | | | | |
| Active, Active, Active | 32 | 35 | 1.0 | 1.0[e] |
| Average, Average, Average | 41 | 30 | 1.6 | 1.2 (0.6–2.7) |
| Inactive, Inactive, Inactive | 70 | 35 | 1.8 | 1.6 (0.7–3.3) |
| All other combinations[f] | 190 | 142 | 1.4 | 1.2 (0.6–2.2) |

[a]Restricted to women age ⩾ 50. [b]Adjusted for age, study area, education, parity, years of use of oral contraceptives years of use of menopausal oestrogens and cigarette smoking. [c]Adjusted for current body mass. [d]Adjusted for nonrecreational activity. [e]Adjusted for recreational activity. [f]Miscellaneous group of women who changed activity levels.

**Table VI** Combined effects of current body mass and lifetime activity at ages 20–29, 30–39 and recently on endometrial cancer risk[a,b]

| *Lifetime (20–29, 30–39, recent)* | *Body mass index* <25 | 25–28 | >28 |
|---|---|---|---|
| *Recreational* | | | |
| Active, Active, Active | 1.0[c] | 0.3 | 2.1 |
| | | (0.1–8.9) | (0.5–8.6) |
| | [21,14] | [6,10] | [10,5] |
| Average, Average, Average | 0.4 | 0.7 | 3.0 |
| | (0.2–1.2) | (0.2–2.2) | (1.0–8.8) |
| | [18,33] | [11,16] | [32,12] |
| Inactive, Inactive, Inactive | 0.7 | 2.4 | 4.1 |
| | (0.3–2.1) | (0.6–9.5) | (1.5–11.3) |
| | [20,29] | [9,6] | [50,15] |
| *Nonrecreational* | | | |
| Active, Active, Active | 1.0[c] | 1.1 | 3.6 |
| | | (0.2–4.7) | (1.1–12.2) |
| | [11,20] | [5,7] | [16,8] |
| Average, Average, Average | 1.1 | 1.5 | 6.9 |
| | (0.3–3.5) | (0.3–8.6) | (2.1–23.1) |
| | [14,17] | [3,6] | [24,7] |
| Inactive, Inactive, Inactive | 2.3 | 2.1 | 4.3 |
| | (0.8–6.9) | (0.6–7.5) | (1.2–13.5) |
| | [30,17] | [13,9] | [27,9] |

[ ] cases, controls; ( ) 95% confidence limits. [a]Restricted to women age ⩾ 50. [b]Adjusted for age, study area, education, parity, years of use of oral contraceptives and years of use of menopausal oestrogens and cigarette smoking. [c]Reference category.

associated with sustained recreational inactivity was limited to women with a body mass of 25 kg m$^{-2}$ or greater. In contrast, risk of endometrial cancer associated with sustained nonrecreational inactivity was present in every stratum of body mass. A similar pattern was observed when this analysis was repeated using recent recreational and nonrecreational activity data (not presented).

*Relation between risk and a single assessment of physical activity*

Finally, we examined the relation between risk according to responses to a single question assessing physical activity (Table VII). After adjustment for confounders, risk was increased among the few women who were recently very inactive (RR = 3.5). Further adjustment for body mass attenuated this association to 2.5 (95% CL = 0.7–8.7). However, no consistent association was observed between risk and either lifetime or average physical activity level measured in this manner.

### Discussion

In summary, we found an association between risk and recent recreational inactivity (active sports and walks or hikes) that was largely explained by the tendency for recreationally inactive women to be overweight or obese. However, a small association between risk and sustained recreational inactivity appeared to persist even after adjustment for body mass. Closer examination showed that this association was restricted to heavier women. We also found associations between risk and recent and sustained nonrecreational inactivity (house cleaning, stair climbing and walking or standing on the job) that were not explained by body mass.

The most likely explanation for excess body mass being largely responsible for the association between recreational inactivity and risk is that physical inactivity contributes to obesity (King & Tribble, 1991), which in turn, increases the risk of endometrial cancer (Kelsey & Hildreth, 1982; Swanson *et al.*, in press). However, it is possible that recreational inactivity is a marker for obesity, that is, heavier women become inactive. In the present study, risk was increased more than 7-fold for women weighing more than 200 lbs compared to women weighing less than 125 lbs (Brinton *et al.*, 1993). Elevated oestrogen levels may account for the incresed risk in obese postmenopausal women, while progesterone deficiency may be more important in obese premenopausal women (Siiteri, 1987; Key & Pike, 1988).

The finding that the association between recreationally inactivity and risk was limited to heavier women may indicate the presence of incomplete adjustment for factors

**Table VII** Relation between endometrial cancer risk and global assessment of physical activity

| *Global activity* | *Cases* | *Controls* | *Adjusted RR*[b] | *Adjusted RR*[b,c] |
|---|---|---|---|---|
| *Recent activity* | | | | |
| Very active | 90 | 84 | 1.0 | 1.0 |
| Fairly active | 125 | 80 | 1.4 | 1.4 (0.9–2.3) |
| Intermediate | 109 | 97 | 1.1 | 1.0 (0.6–1.6) |
| Fairly inactive | 58 | 32 | 1.5 | 1.1 (0.6–2.0) |
| Very inactive | 17 | 4 | 3.5 | 2.5 (0.7–8.7) |
| *Average lifetime activity* | | | | |
| Q1 Active | 73 | 59 | 1.0 | 1.0 |
| Q2 | 39 | 39 | 0.8 | 0.7 (0.4–1.4) |
| Q3 | 120 | 89 | 1.5 | 1.3 (0.8–2.2) |
| Q4 | 93 | 75 | 1.2 | 1.0 (0.7–1.7) |
| Q5 Inactive | 80 | 35 | 1.9 | 1.4 (0.7–2.6) |
| *Lifetime activity (ages 20–29, ages 30–39, recent)*[a] | | | | |
| Always very active | 66 | 61 | 1.0 | 1.0 |
| Always fairly active | 43 | 21 | 1.8 | 1.5 (0.7–3.2) |
| Always average | 48 | 39 | 1.1 | 1.1 (0.6–2.1) |
| Always fairly inactive | 10 | 3 | 1.9 | 1.7 (0.4–7.7) |
| Always very inactive | 1 | 1 | 1.7 | 1.9 (0.1–41.5) |
| All other combinations[d] | 171 | 117 | 1.4 | 1.1 (0.7–1.9) |

[a]Restricted to women age ⩾ 50 years. [b]Adjusted for age, study area, education, parity, years of use of oral contraceptives, years of use of menopausal oestrogens, and cigarette smoking. [c]Adjusted for current body mass. [d]Miscellaneous group of women who changed activity levels.

related to obesity. For example, the higher risk of endometrial cancer in active heavy women compared to inactive heavy women have less body fat than inactive heavy women, even for the same level of body mass. On the other hand, one might expect adverse effects of inactivity to be most apparent among overweight and obese women if, as has been suggested (Levi *et al.*, in press), physical activity increases the metabolism of endogenous oestrogens to less hazardous forms (Levi *et al.*, in press).

Nonrecreational inactivity was associated with an increase in risk that was not accounted for or modified by excess body mass. This observation suggests that physical inactivity may influence risk through pathways that do not directly involve obesity. A recent study that examined serum levels of oestrogens among postmenopausal women lends some support to this hypothesis (Cauley *et al.*, 1989). After taking differences in body mass into account, Cauley and colleagues (1989) observed that serum oestrogen levels were lower among more active women than among less active women. However, it is not obvious why recreational exercise would alter endometrial cancer risk primarily through its effects on body mass whereas nonrecreational activity would operate through a different mechanism. However, we note that nonrecreational activities tend to be maintained at moderate intensity whereas recreational exercise tends to be intermittent at high intensity.

There are several limitations of this study which need to be discussed. First, there is a possibility that the lower activity among cases reflects a change in response to symptoms of overt or preclinical illness. We attempted to address this issue by excluding information on activity during the age decade preceding the one in which endometrial cancer was diagnosed and a comparable time period in controls. In addition, the study of endometrial cancer may be less problematic than other cancers because in most instances the disease is detected at early stage. Finally, results were similar to those presented when we excluded women with later stage disease (data not presented).

Another limitation of this study is the low interview response rate among the controls. If controls who were physically inactive were disproportionately less likely to be interviewed than cases, this could result in a spurious association between physical inactivity and endometrial cancer risk. It is somewhat reassuring, however, that findings from this study with respect to generally accepted endometrial cancer risk factors (Brinton *et al.*, 1993), including body mass index, are similar to those reported in previous studies (Kelsey & Hildreth, 1982).

A third limitation of this study is that the assessment of physical activity was based only on a brief interview instrument, covering several selected aspects of physical activity. Because we relied on self-reported data and did not collect detailed information on the frequency of participation in specific activities, it is likely that some women have been misclassified with respect to activity level. However, this type of misclassification would tend to obscure an underlying association rather than produce a spurious one.

A fourth more serious concern is that the association between nonrecreational activity and risk may be due to confounding by unmeasured lifestyle factors. Although differences in known risk factors, such as cigarette smoking, nulliparity and hormone use, did not account for the results for nonrecreational activity we cannot dismiss the role of other behaviours. One component of this measure was whether women walked or stood on their jobs. However, since the women classified as inactive on this measure included retired individuals, those who never worked outside the home, as well as those who were truly inactive on their jobs, it is difficult to assess the meaning of the association. One other component of this measurse was frequency of house cleaning, which could reflect a variety of social class factors. It is, however, reassuring that the remaining component of nonrecreational activity, namely climbing stairs, did appear to influence risk, especially since this aspect of activity has been found to be associated with risk of other disease outcomes, such as diabetes (Helmrich *et al.*, 1991). Furthermore, some support for our findings for nonrecreational activity are provided by an analysis of cancer incidence data from the US National Health and Nutritional Survey cohort (Albanes *et al.*, 1989). Albanes and colleagues (1989) found that nonrecreational inactivity was associated with a modest increase in risk of all cancers combined, after adjustment for body mass and possible confounders. In contrast, recreational exercise was unrelated to risk.

We were unable to demonstrate a convincing association between risk and activity level measured by a single question. Although women who reported that they were recently very inactive were at increased risk, we have concerns about our ability to adequately adjust for potential confounders because few women reported being very inactive. Furthermore, average and lifetime perceived activity measured in this manner were unrelated to risk. We are inclined to conclude that perceived activity level, because of its subjective nature, may be more prone to bias and misclassification than data based on frequency of specific activities.

Interest in the possibility that endometrial cancer risk might be elevated among physically inactive women was generated when Frisch and colleagues (1985), in a cross-sectional study of college alumni, found that women who had engaged in college athletics had a lower prevalence of reproductive cancer (cervix, endometrium, ovary and vagina combined). Few studies have specifically examined the relation between physical activity and endometrial cancer, and the results have been conflicting. In a study conducted in China, Shu and colleagues (in press) found that occupational inactivity among non-retirees was associated with an increased risk of endometrial cancer but that leisure time inactivity was associated with decreased risk. Adjustment for body mass and possible confounders did not alter their results. After adjustment for body mass and other confounders, a study conducted in Italy and Switzerland (Levi *et al.*, in press) found that endometrial cancer cases were less likely to be physically active than hospital controls. Infrequent recent participation in certain activities, including sports and leisure, occupational activity and house keeping were associated with increased risk whereas infrequent participation in walking or hikes and climbing stairs was not.

In summary, our data indicate that inactive women may be at increased risk of endometrial cancer by virtue of their tendency to be overweight or obese. There was also a suggestion in our data that physical inactivity *per se* may increase endometrial cancer risk, but this should be considered tentative because of some inconsistencies within our own dataset and across published studies on this topic. Further confirmation of this finding is needed in studies that collect detailed information on different dimensions of physical activity, including the intensity with which an individual engages in an activity and actual time involved in exertion. It would also be useful to establish whether there are long term effects of moderate physical activity on circulating hormone levels.

### References

ALBANES, D., BLAIR, A. & TAYLOR, P.R. (1989). Physical activity and risk of cancer in the NHANES I population. *Am. J. Public Health*, **79**, 744–750.

ASHWELL, M., COLE, T.J. & DIXON, A.K. (1985). Obesity: new insight into the anthropometric classification of fat distribution by computed tomography. *BMJ* 290, 1692–1694.

BRESLOW, N.E. & DAY, N.E. (1980). *Statistical Methods in Cancer Research: The Analysis of Case-Control Studies*. IARC Scientific Publications: Lyon, France.

BRINTON, L.A., BERMAN, M.L., MORTEL, R., TWIGGS, L.B., BARRETT, R.J., WILBANKS, G.D., LANNOM, L. & HOOVER, R.N. (1993). Reproductive, menstrual and medical risk factors for endometrial cancer: results from a case-control study. *Am. J. Obstet. Gynecol.*, **81**, 265–271.

CAULEY, J.A., GUTAI, J.P., KULLER, L.H., LEDONNE, D. & POWELL, J.G. (1989). The epidemiology of serum sex hormones in postmenopausal women. *Am. J. Epidemiol.*, **129**, 1120–1131.

FRISCH, R.E., WYSHAK, G., ALBRIGHT, N.L., ALBRIGHT, T.E., SHIFF, I., JONES, K.P., WITSHI, J., SHIANG, E., KOFF, E. & MARGUGLIO, M. (1985). Lower prevalence of breast cancer and cancers of the reproductive system among former college athletes compared to non-athletes. *Br. J. Cancer*, **52**, 885–891.

HELMRICH, S.P., RAGLAND, D.R., LEUNG, R.W. & PAFFENBARGER, R.S. Jr (1991). Physical activity and reduced occurrence of non-insulin-dependent diabetes mellitus. *N. Engl. J. Med.*, **325**, 147–152.

KELSEY, J.L. & HILDRETH, N.G. (1982). *Breast and Gynecologic Cancer Epidemiology*. CRC Press: Boca Raton.

KEY, T.J.A. & PIKE, M.C. (1988). The role of oestrogens and progestagens in the epidemiology and prevention of breast cancer. *Eur. J. Cancer Clin. Oncol.*, **24**, 29–43.

KING, A.C. & TRIBBLE, D.L. (1991). The role of exercise in weight regulation in nonathletes. *Sports Med.*, **11**, 331–349.

LEVI, F., LA VECCHIA, C., NEGRI, E. & FRANCESCHI, S. (1993). Selected physical activities and risk of endometrial cancer. *Br. J. Cancer*, (in press).

POTISCHMAN, N., SWANSON, C.A., BRINTON, L.A., MCADAMS, M., BARRETT, R.J., BERMAN, M.L., MORTEL, R., TWIGGS, L.B. & WILBANKS, G.D. (1993). Dietary associations in a case-control study of endometrial cancer. *Cancer Causes and Control*, **4**, 239–250.

SIITERI, P.K. (1987). Adipose tissue as a source of hormones. *Am. J. Clin. Nutr.*, **45**, 277–282.

SCHWARTZ, S.M., THOMAS, D.B. AND THE WORLD HEALTH ORGANIZATION COLLABORATIVE STUDY OF NEOPLASIA AND STEROID CONTRACEPTION (1989). *Cancer*, **64**, 2487–2492.

SHU, X.O., HATCH, M.C., ZHENG, W., GAO, Y.T. & BRINTON, L.A. (1993). Physical activity and risk of endometrial cancer. *Epidemiology*, (in press).

SWANSON, C.A., POTISCHMAN, N., WILBANKS, G.D., TWIGGS, L.B., MORTEL, R., BERMAN, M.L., BARRETT, R.J., BAUMGARTNER, R.N. & BRINTON, L.A. (1993). Risk of endometrial cancer in relation to contemporary and past body size. *Cancer Epidemiology, Biomarkers and Prevention*, (in press).